# Editorial: Bridging discovery and translation in novel biomarkers and big data-based biomedical studies for cancer management

**DOI:** 10.3389/fmolb.2025.1620512

**Published:** 2025-05-22

**Authors:** Lin Zhang, Weiye Qian, Qingyu Luo

**Affiliations:** ^1^ The School of Public Health and Preventive Medicine, Monash University, Melbourne, VIC, Australia; ^2^ Department of Medical Research, Suzhou Industrial Park Monash Research Institute of Science and Technology, Suzhou, China; ^3^ Department of Medical Oncology, Dana-Farber Cancer Institute, Harvard Medical School, Boston, MA, United States; ^4^ One Patient One Cure, Boston, MA, United States

**Keywords:** noval biomarkers, big data, biomedical studies, cancer diagnosis, cancer management

Cancer remains one of the most formidable challenges in modern medicine. The discovery of novel biomarkers and the application of big data-based biomedical studies have emerged as powerful tools to enhance our understanding, diagnosis, and management of this complex disease. As a testament to this progress, we present a collection of 21 articles published for the *Novel Biomarkers and Big Data-Based Biomedical Studies in Cancer Diagnosis and Management* research topic in Frontiers in Molecular Biosciences.

This collection of papers highlights the critical role of biomarker discovery and research in advancing cancer treatment. Al Shareef et al. investigated the prognostic value of Dickkopf-3 (Dkk3), TGFB1, and ECM-1 in prostate cancer and found that the decreased expression of DKK3 and the increased expression of TGFB1 were closely related to disease progression and poor prognosis, highlighting their potential as prognostic indicators. Zhu et al. identified TUBB as a robust biomarker through comprehensive pan-cancer analysis. TUBB was consistently overexpressed in multiple tumor types and was significantly associated with prognosis, immune invasion, and chemosensitivity. Meanwhile, Torres-Llanos et al. proposed the MIR4435-2HG as a regulatory biomarker predicting treatment response and survival in pediatric B-cell ALL, with its overexpression being linked to positive minimal residual disease and increased risk of poor outcomes. These findings highlight the prevalence and specificity of molecular biomarkers across different cancer types.

This paper collection also spans a broad range of cancer types, demonstrating the diversity of current biomarker discoveries. Xie et al. discovered novel junctional genes associated with the survival of patients with lung adenocarcinoma (LUAD), establishing a risk model that integrates clinical features for improved prognosis prediction. Fan et al. confirmed that elevated AVEN expression was associated with tumor progression and poor survival rate in LUAD, and they established a robust AVEN-derived prognostic model, which was validated in an external cohort. Chen et al. found that low expression of circadian gene period2 (PER2) in hepatocellular carcinoma (HCC) was associated with immune infiltration and adverse clinical features, highlighting its diagnostic and prognostic potential. Zhang et al. constructed a three-gene marker based on the E2F, which can predict the prognosis, immune evasion, and drug sensitivity of HCC, suggesting its clinical application in personalized treatment. Chen et al. identified risk markers of endoplasmic reticulum stress-related genes in pancreatic cancer, revealing associations with poor survival rate and changes in the immune microenvironment.

The integration of big data analytics and artificial intelligence into cancer research is another cornerstone of this research topic. Jarwal et al. combined single-cell transcriptomics and deep learning to accurately classify head and neck squamous cell carcinoma (HNSCC) and human papillomavirus (HPV) status, providing a non-invasive diagnostic tool. Lei et al. developed a six-gene prognostic model based on coagulation-related genes, which can stratify breast cancer patients according to survival outcomes and guide treatment strategies, including immunotherapy and chemotherapy. Panthi et al. established radiological models using longitudinal DCE-MRI features to predict the early treatment response of triple-negative breast cancer and promote personalized treatment decisions. Liu et al. conducted a meta-analysis demonstrating that CT-based radiomics provides reliable diagnostic performance in predicting lymph node metastasis of esophageal cancer, highlighting its clinical value in staging.

Several studies emphasize the importance of translational research and clinical applications. Liu et al. explored the application of shear wave elastography in 60 rectal cancer patients to predict the pathological complete response (ypT0) stage after neoadjuvant therapy in rectal cancer, demonstrating that corrected elasticity parameters significantly improved prediction of the ypT0 stage compared with traditional ultrasound, thereby supporting a watch-and-wait strategy. Similarly, Wang et al. conducted a systematic review and meta-analysis of 44 studies (n = 2430) on neoadjuvant immunotherapy protocols in non-small cell lung cancer. Their research results show that compared with immunotherapy alone, chemotherapy combined with immunotherapy significantly increased the rates of major and complete pathological responses, and the three treatment cycles provided a good balance between efficacy and adverse events.

This topic also includes new advances in breast cancer research and treatment through multiomics and clinical analyses. Feng et al. conducted a Mendelian randomization (MR) analysis to study the potential causal relationship between the oral microbiome and the risk of seven major cancers, including breast cancer, and identified microbial genera that have different effects on cancer susceptibility. In a supplementary study, Feng et al. conducted 16S rRNA gene sequencing on oral, intestinal, and breast tissue samples from patients with different pathological subtypes of breast cancer and found that microbial patterns varied across sample types and cancer subtypes. Tutzauer et al. investigated gene expression profiles in primary tumors and distant metastases in metastatic breast cancer patients. Five distinct gene expression subtypes were identified among metastases, indicating that the expression of androgen receptors at the primary and metastatic sites is a powerful prognostic marker for progression-free survival. Wang et al. conducted an comprehensive proteomic and transcriptomic analysis using proteome/transcriptome-wide association studies and MR, identifying several plasma proteins (such as PEX14, CTSF, and SNUPN) with causal links to breast cancer risk, which also showed subtype-specific patterns. In another study, Feng et al. evaluated the actual impact of aggressive locoregional surgery, including infraclavicular and supraclavicular lymph node dissection, in breast cancer patients with ipsilateral supraclavicular lymph node metastasis. They found that more extensive axillary dissection improved overall and disease-free survival, although supraclavicular dissection alone did not add further benefit. Finally, Li et al. reported a novel copy number variant in the APC gene in a family with familial adenomatous polyposis, while another study by Li et al. examined lymphoma patients with COVID-19, highlighting the delayed viral clearance and its potential association with compromised immune function in malignancy.

The articles collected in this research feature represent a comprehensive and impactful effort to advance cancer diagnosis, prognosis, and treatment through biomarker discovery and big data-driven approaches. By integrating molecular analysis, artificial intelligence, imaging technologies, and translational research, these works highlight how modern research shapes personalised cancer treatment. From the identification of prognostic genes and immune-related markers to the application of deep learning and radiomics, each study has provided new insights with potential clinical implications. These include rigorous clinical evaluations, such as shear wave elastography in rectal cancer and meta-analyses of immunotherapy cycles in lung cancer, highlighting the translational focus. In conclusion, these contributions not only deepen our understanding of cancer biology but also lay the foundation for more precise, individualised, and effective treatment strategies for various tumour types.

